# PPTPS: Building privacy-preserving auditable service with traceable timeliness for public cloud storage

**DOI:** 10.1371/journal.pone.0276212

**Published:** 2022-10-28

**Authors:** Li Li, Xiao Lan, Mali Chen, Ting Luo, Li Chen, Yangxin Wang, Yumeng Chen

**Affiliations:** 1 School of Artificial Intelligence, Chongqing University of Education, Chongqing, China; 2 Cybersecurity Research Institute, Sichuan University, Chengdu, Sichuan, China; 3 School of Mathematics and Big Data, Chongqing University of Education, Chongqing, China; Northeastern University at Qinhuangdao, CHINA

## Abstract

Many works are designed to improve efficiency or enhance security and privacy of publicly-auditable cloud storage. However, building timeliness for cloud storage has not been well studied. Few works presented time-sensitive cloud storage and only focused on specific issues, such as the earliest creation time of files or resistance against a procrastinating auditor. Therefore, there leaves an absence of building traceable timeliness for publicly-auditable cloud storage. In this paper, we propose a solution PPTPS to build Privacy-Preserving auditable service with Traceable timeliness for Public cloud Storage. First, we use the security properties of the blockchain to provide a time-stamp for each phase. It enables the timeliness of cloud storage. Second, we construct efficient publicly-verifiable cloud storage. Third, a customized random mask solution is proposed to prevent privacy leakage during the auditing phase. Moreover, we formally proved the security of PPTPS. At last, experimental results demonstrate that PPTPS is economically sound and technically viable.

## 1 Introduction

Cloud storage service offers customers an efficient and economical way to store data and work together [1–3]. Many companies prefer to outsource data to cloud storage service providers, e.g., Dropbox, Amazon, and TortoiseSVN. However, data on the cloud often risks accidental loss or corruption from casual mistakes or hackers’ attacks [4]. Therefore, researchers have proposed publicly auditable solutions focusing on data integrity verification to address the data corruption problem in the past few years. Moreover, the cloud service providers need to preserve the outsourced data stably for archiving and keep track of the critical activities, such as outsourcing, auditing, proving, and verifying activities. Therefore, the traceable timeliness of data on the cloud is essential except for the integrity audit. For example, for effectively controlling COVID-19, tracing the earliest creation time of an infected patient’s data is critical. Also, in the online patent application, the timeliness of the patent determines the fairness of conclusions in judgments and dispute resolutions. Nevertheless, traditionally time-stamping schemes relied on a central service provider. Once this provider is compromised, attackers can arbitrarily modify time stamps. Hence, a time-stamping solution is desirable to be free from the trust of the third time-stamping provider. Therefore, it is urgent to build traceable management for publicly-auditable cloud storage.

Overall, there are two limitations of previous works as follows. The first limitation is the absence of traceable timeliness for data on the cloud. However, few solutions are proposed to address the specific time-sensitive issues of cloud storage, e.g., resisting procrastinating auditors or determining the earliest data creation time. Moreover, the method to build the timeliness to provide traceable management for data on cloud storage is not well investigated. The second limitation is the existing publicly-auditable storage systems are not so efficient and have an information leakage problem during auditing. Lightweight publicly-auditable cloud storage with privacy-preserving enhancement is favorable in practice.

The first challenge is how to build the timeliness for cloud storage. The most popular solution is to make a time-stamp for each phase. Nevertheless, traditional solutions with trusted time-stamp service providers [5, 6] suffered from a single point of failure problem. Cao et al. utilized blockchain hashes to enable the timeliness of EHRs [7]. However, it cannot provide sufficient security by only using the latest block hash value due to the possibility of a blockchain fork. To cope with this problem, we adopt a fixed number of successive block hashes with the current block height [8], which are integrated into a block to provide an accurate time-stamp for each phase. Besides, these successive block hashes, the current block height, and other important information are recorded as entries for traceability on the cloud.

The second challenge is designing lightweight, privacy-preserving cloud storage without sacrificing security. To achieve more efficiency, we customize publicly-verifiable cloud storage through lightweight cryptographic tools and employ the random mask technique to enhance the privacy-preserving property during the audit phase.

In what follows, this paper achieves five design goals.

- **Public audit**. In contrast to the private audit, the public audit enables any party to check the data integrity on the cloud for various applications.
- **Traceability**. The timeliness is exhibited explicitly. We can get an accurate time-stamp of data in each phase. We can check the operations through the time-stamp of log entries stored on the cloud.
- **Auditing correctness**. Any data with corruption can pass the verification of an auditor with negligible probability.
- **Privacy-preserving**. Data content cannot be leaked to the auditor during the auditing phase. The security and privacy of data should be well protected.
- **Efficiency**. The computation and communication costs are acceptable in practice. Lightweight cryptographic tools are adopted.

This paper makes four main contributions as follows.

1. We propose a method to build traceable management for data on the cloud. For each phase of cloud storage, the corresponding information, the current block height, and a fixed number of successive block hashes are stored on the cloud and integrated into blockchain transactions. The timeliness based on the blockchain’s security properties explicitly provides us with the operation time of data for traceability.
2. We design a lightweight privacy-preserving publicly-auditable cloud storage system. We employ lightweight cryptographic tools rather than heavy ones to construct this system based on the DL assumption. Additionally, we enhance the privacy-preserving property of the auditing process with a customized random mask method and ensure that the auditor cannot obtain any data from the information.
3. We formally prove security of PPTPS and further give the security analysis in four distinct aspects. In addition, we conduct extensive experiments to demonstrate PPTPS’s performance superiority to other works regarding computation and communication costs.

The construction of our article is organized as follows. Section 2 reviews the related works and Section 5 gives the problem statement. Background knowledge is included in Section 3 as preliminaries. Section 6 describes our scheme in detail and Section 7 gives the security analysis. Experimental results are demonstrated in Section 8. Finally, Section 9 concludes our article.

## 2 Related work

### 2.1 Secure auditable cloud storage

Since Ateniese et al. presented Proof of Data Possession (PDP) [9] and Juels et al. proposed Proof Of Retrievability (POR) [10], many works have emerged in the field of cloud storage [11–16]. First, Shacham et al. presented a solution SWP and adopted BLS signatures to support verifiability in [11]. Later, Wang et al. proposed a privacy-preserving publicly-auditable cloud storage system called PP-SWP by leveraging the random masking technique [12] to enhance the privacy-preserving property. Later, Chen et al. constructed a network coding-based public cloud storage system [13]. Both these two works support public verifiability but suffer from efficiency. Zhang et al. also used the DL assumption to support personal verification in [16]. However, these schemes are incapable of traceability. Tian et al. did not use the computationally complicated bilinear pairings for greater efficiency and used the DL assumption to construct a publicly-auditable cloud storage system [17].

### 2.2 Privacy-preserving cloud storage

Generally, a cloud storage system includes four phases [18–25]. First, the public and private keys for the cloud storage system are generated during the key generation phase. Next, data blocks with tags are outsourced to the CSP in the outsourcing phase. Moreover, an auditor generates a query and sends it to the CSP in the query phase. Then, in the proof generation phase, the CSP computes the corresponding proof and sends the proof to the auditor. Finally, the auditor validates the proof and checks whether the data on the CSP is intact or not in the verification phase. Moreover, to prevent the auditor from collecting the information during the auditing phase to derive the users’ data, some privacy-preserving techniques in distinct aspects are adopted, such as the random mask, the homomorphic secret sharing, and ring signature [12, 26, 27].

### 2.3 Blockchain-based retroactive storage

The blockchain enables medical centers to be built on different platforms to share EHRs without a third authority. In [7], a cloud-assisted eHealth system called CASES was proposed to resist illegal modifications of outsourced EHRs. In CASES, the auditor with a specific warrant can audit the outsourced EHRs. Hence, it did not support public verifiability well. Soon later, to construct a cloud storage system against procrastinating auditors, Zhang et al. proposed CPVPA, a public integrity verification method for cloud storage [8], by using the security properties of the blockchain. It enabled the detection of procrastinating auditors in time and allowed user checks. In [28], the nonces in a blockchain are used to construct unpredictable challenge data. Therefore, the forging auditing results from malicious TPAs can be prevented. In [29], an accurate time stamping scheme, Chronos^+^, to judge the earliest creation time of a file was proposed by leveraging the blockchain’s chain growth property. However, these two works focused on specific storage problems, such as resisting procrastinating auditors and determining the earliest creation time of a file. Therefore, the solutions cannot support traceable management for storage straightforwardly. Later, Kim et al. presented a secure protocol for cloud-assisted EHR systems using the blockchain [30]. It comprised six phases: registration, authentication, smart contract uploading, EHR storing, EHR requesting, and log transaction uploading. However, these complicated phases brought a heavy burden regarding computation and communication costs. In a word, there is no traceable storage management concept in previous works. In [31], Xie et al., the authors applied smart contracts instead of untrusted third-party auditors to improve the reliability and stability of audit results. In [32], Zhang et al., the authors used the blockchain to record the interactions among users, service providers, and organizers in the data auditing process as evidence. Moreover, the smart contract was employed to detect service disputes.

For ease of explanation, the comparisons with previous works in different aspects are described in Table 1 as follows.

**Table 1:** Comparisons with previous works.

Works	Traceability	All-phases management	Public audit	Privacy preserving in the audit phase
SWP [11]	×	×	✓	×
PP-SWP [12]	×	×	✓	×
SCS-NC [13]	×	×	✓	×
SCS-DLP [16]	×	×	×	×
CASES [7]	✓	×	×	✓
CPVPA [8]	✓	×	✓	×
Chronos^+^ [29]	✓	×	×	×
CPIGS [38]	×	×	✓	✓
PPTPS	✓	✓	✓	✓

## 3 Preliminaries

### 3.1 Ethereum

The blockchain is a growing list of chained blocks. The blockchain is resistant to data modification. Once recorded into the blockchain, anyone cannot alter the data in the block retroactively without controlling more than the 51% hash rate. The blockchain includes two types: the public blockchain and the private blockchain. As one type of the most popular public blockchain, Ethereum is a network architecture composed of decentralized nodes called Ethereum nodes. Anyone with sufficient computer hardware can join the Ethereum network as a node and contribute computing power to earn block mining rewards. Up to 2022/09/17, about 10,490 Ethereum nodes were distributed worldwide.

Each block of Ethernet comprises two parts: header and body. The body includes the list of transactions. Moreover, the block header is more complex, containing the previous block’s hash, a time-stamp, mining difficulty, and other related parameters. Ethereum has two types of accounts: contract and externally owned accounts. A contract account has an ether balance, which stores the contract code that determines the ether change in the account.

The mining process is to pack the validated pending transactions into new blocks and use computing power to calculate the nonce value. The first miner who finds a nonce value and broadcasts this value will be rewarded with a fee (Gas) deriving from the transactions within the block.

As a new block has been created, all nodes need to synchronize the block. Once all nodes accept the block, previously uncounted blocks will expire, and each node will recreate a block. The block-out time for each block is about 10s. As the computing power of the whole network keeps changing, the generation time of each block will be shortened as the computing power increases and lengthened as the computing power decreases [33].

On Ethereum, the blockchain height *t* denotes the current amount of blocks in the blockchain. Moreover, there are two properties of the blockchain as our fundamental to construct PPTPS [34–36].

- *ϕ*-chain consistency: At any two rounds during an executing process, any two honest parties of the blockchain can differ only in the last *ϕ* blocks. In Ethereum, the number of blocks is set as *ϕ* ≥ 12.
- Chain growth: During any time interval, the number of blocks chained into the blockchain is deterministic. Besides, in a given term, the block height increases steadily.

### 3.2 Bilinear maps

$G_{1}$ and $G_{2}$ are two multiplicative cyclic groups with large prime order *p*. *g*_1_ is the generator of $G_{1}$, and *g*_2_ is the generator of $G_{2}$. $e:G_{1}\times G_{2}\to G_{T}$ is the function that maps pairs of elements in $G_{1}$ and $G_{2}$ to elements in the cyclic group $G_{T}$ with the prime order *p*. The unique features of bilinear maps are listed as follows:

- Bilinear mapping: for $\forall u\in G_{1}$, $\forall v\in G_{2}$, $\forall a,b\in Z_{p}^{*}$, the equation *e*(*u*^*a*^, *v*^*b*^) = *e*(*u*, *v*)^*ab*^ holds.
- Non degenerate: $\exists u,v\in G_{1}$, such that *e*(*u*, *v*) ≠ 1.
- Computional: for $\forall u,v\in G_{1}$, *e*(*u*, *v*) is computational in the polynomial time.

### 3.3 Complexity assumption

**Discrete logarithm problem**. In [37], the discrete logarithm problem is formally defined. Given a large prime *p*, a cyclic group G with the order *p*, a generator g∈G, and a random value r∈G, it is computationally infeasible to compute $a\in Z_{p}$ satisfying *r* = *g*^*a*^.

**The CDH assumption**. Given *g*, *g*^*s*^, *g*_0_ ∈ *G*_1_, for unknown $s\in Z_{q}^{*}$, no probabilistic polynomial-time algorithm can compute $g_{0}^{s}$ with non-negligible advantage.

## 4 Outline of publicly-auditable cloud storage

Generally, publicly-auditable cloud is composed of six algorithms including Setup, KeyGen, Outsource, Query, Prove, and Verify.

- Setup: With the security parameter, the data owner outputs the system parameters.
- KeyGen: This algorithm comprises two algorithms. In the algorithm SecretKeyGen the data owner outputs the private key *sk*. In the algorithm PublicKeyGen the data owner outputs the public key *pk*.
- Outsource: For each block, the data owner inputs the private key and generates a tag.
- Query: The auditor generates some random indexes with random values as an audit query and sends the audit query to the CSP.
- Prove: The CSP generates the proof for the query and returns the proof to the auditor.
- Verify: Upon receiving the proof, the auditor verifies the proof and outputs the result to demonstrate whether data on the CSP are intact or not.

## 5 Problem statement

First, we introduce the system model for the whole framework. Then we define the adversary model of PPTPS.

### 5.1 System model

**Fig 1:**
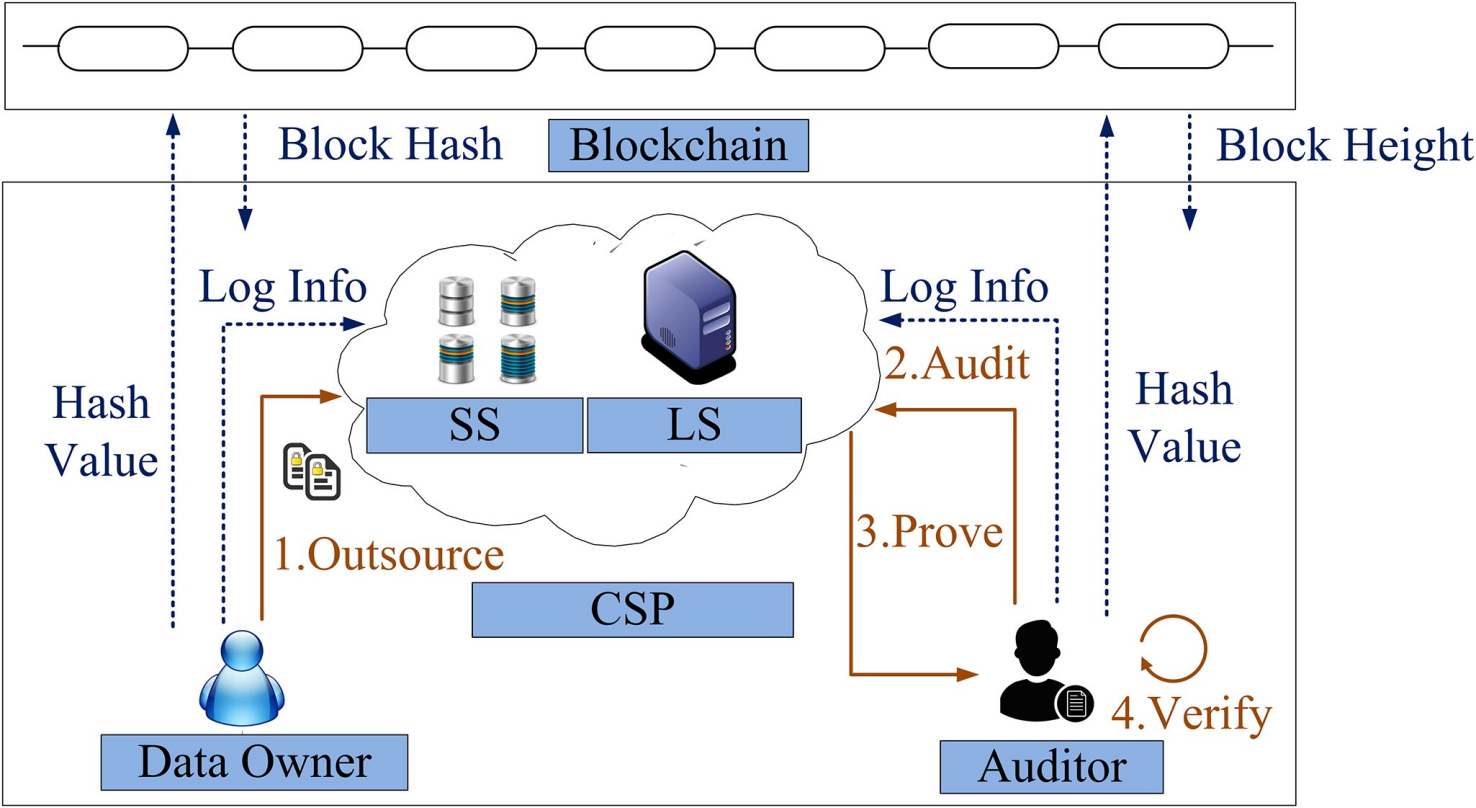
Framework of PPTPS.

As shown in Fig 1, six entities are included in PPTPS. The solid line represents the process, and the dotted line represents the message delivered.

- The Cloud Server Provider (CSP): In PPTPS, CSP includes two parts. One part is a storage server that stores data and generates integrity proof traditionally. The other part is a log server. The log server is responsible for recording the corresponding time-stamp information.
- The Data Owner: The data owner is responsible for outsourcing the data to the CSP.
- The Auditor: The auditor generates an audit query and verifies the proof returned from the CSP.
- Blockchain: The blockchain provides the time-stamp functionality for PPTPS. In each phase, the blockchain outputs the current block height for other parties and then receives the transactions generated by other parties.

We describe Fig 1 briefly. First, the data owner outsources the data to the storage server of CSP. Subsequently, an auditor generates an audit query for the storage server. Then the storage server generates the corresponding proof. Later, the auditor verifies the proof to check whether the data on the storage server is intact or not. The data privacy is not leaked to the auditor during the audit phase. Besides, the log server of CSP records the log entries in each phase of PPTPS for traceability. The blockchain provides each phase’s hash values and the current block height. We build traceable management for a privacy-preserving publicly-auditable cloud storage system through the log entries and the blockchain.

### 5.2 The correctness definition

Here we present the correctness definition, which will be used in the subsequent sections.

**Definition 1 (Correctness)**: *For the key K* *and EHRs F*, *let* *F*′ = Outsource, *q* = Query, *and* Γ = Prove, *if the algorithm* Verify(Γ *K*) *outputs true with all but negligible probability, then PPTPS satisfies correctness*.

### 5.3 Adversary model

In the adversary model, we categorize the threats into two types: internal and external adversaries. Furthermore, the internal adversary includes the malicious CSP, the procrastinating auditor, and the malicious data owner. The adversaries and their attack abilities are described as follows.

**The external adversary**: This adversary attempts to fore the tag of the block and breaks the security of a cloud storage system. Referring to [38], we present a generic framework for a game $\text{Game}I(C,A_{I})$, where a challenger is denoted as C and an adversary is denoted as $A_{I}$. In $\text{Game}I(C,A_{I})$, there are three algorithms in the following.

- Setup: The challenger C runs this algorithm to get the system parameters. Then the challenger C sends the system parameters to the adversary $A_{I}$.
- Queries: The adversary A adaptively generates different queries to the challenger C. Then the challenger C responds to the queries to the adversary $A_{I}$.
  - Hash Query: The adversary $A_{I}$ adaptively asks hash queries to the challenger C. C responses the hash values to the adversary $A_{I}$.
  - Key Query: The adversary $A_{I}$ adaptively asks key queries to the challenger C. C executes the algorithm SecretKeyGenand PublicKeyGento obtain the private key and the public key, respectively. C returns the private key and the public key to $A_{I}$.
  - Tag Query: The adversary $A_{I}$ adaptively chooses the block *d* and sends *i* to C for querying the tag for this block.
- Forge: The adversary $A_{I}$ outputs a forged tag *s*′ for *d*′.

If the forged tag *s*′ for *d*′ is valid, then $A_{I}$ wins the game.

**Definition 2**: *If* $A_{I}$ *wins* $\text{Game}I(C,A_{I})$ *with negligible probability, then the probability to forge a block tag is negligible*.

From Definition 2, we obtain that without the correct private key, the adversary $A_{I}$ cannot forge the tag of its corresponding block.

**The malicious CSP**: Since data and tags are stored on the CSP, we must consider the problem that if the data is corrupted, the CSP will try to cheat the auditor that the data is still kept intact. We consider the malicious CSP as the adversary $A_{II}$. In the following $\text{Game}II(C,A_{II})$, we focus on the problem that whether the adversary $A_{II}$ can forge a valid proof on corrupted data and pass the verification. In $\text{Game}II(C,A_{II})$, there are four algorithms in the following.

- Setup: The challenger C runs this algorithm to get the system parameters and the private key. Then the challenger C keeps the private key and sends the system parameters to the adversary $A_{II}$.
- SecretKey Query: The adversary $A_{II}$ adaptively asks the secret key query to the challenger C. C executes the SecretKeyGen algorithm to obtain the secret key and sends the secret key to $A_{II}$.
- PublicKey Query: The adversary $A_{II}$ adaptively asks the public key query to the challenger C. C executes the PublicKeyGen algorithm to obtain the public key and sends the public key to $A_{II}$.
- Tag Query: The adversary $A_{II}$ adaptively chooses the block *d* and sends *d* to C for querying the tag for this block. C runs the algorithm Outsource and generates the tag for the block *d*. Finally C returns back the tag to $A_{II}$.
- Forge: $A_{II}$ forges the proof and sends the proof to C. If the proof can pass the verification without correct data, $A_{II}$ wins the $\text{Game}II(C,A_{II})$.

**Definition 3**: *If for the adversary* $A_{II}$, $A_{II}$ *wins the game* $\text{Game}II(C,A_{II})$ *with negligible probability, then the probability to forge a valid proof without correct data is negligible*.

**The procrastinating auditor**: The malicious auditors can perform the following attacks. First, a malicious auditor forges an entry that passes the verification successfully. Second, a malicious auditor forges an entry at the block height *t*_1_ but claims that the entry is generated at the block height *t*_2_, where *t*_2_ > *t*_1_.

## 6 Our scheme

### 6.1 Construction of PPTPS

- Setup: With the security parameter *κ*, the following parameters are generated including a prime *p* with bit length at least *κ*, two multiplicative cyclic groups $G_{1}$(a generator for $G_{1}$ is *g*) and $G_{2}$ with the order *p*, and a secure hash function $H:{\left\{0,1\right\}}^{*}\to G_{1}^{*}$. The file *F* with the name *fn* is divided into *m* blocks and each block is denoted as *d*_*i*_, where *i* = 1, 2, …, *m*. The system parameters for PPTPS are $\left(p,G_{1},G_{2},g,H\right)$. Besides, the data owner, CSP, and the auditor create externally owned accounts *A*_*D*_, *A*_*C*_, and *A*_*U*_ in Ethereum, respectively.
- KeyGen: In the algorithm SecretKeyGen, the data owner randomly chooses $x_{1},x_{2},\ldots ,x_{m}\in Z_{p}^{*}$ as the private key *sk*. In the algorithm PublicKeyGen, the data owner computes $y_{1}=g^{x_{1}},y_{2}=g^{x_{2}},\ldots ,y_{m}=g^{x_{m}}$ as the public key *pk*.
- Outsource: For each block *d*_*i*_, using the private key *x*_*i*_, the data owner computes a tag $s_{i}=g^{d_{i}}\cdot H{\left(w\right)}^{x_{i}}$, *w* = *i*‖*fn*. Then, the data owner extracts the block height *t*_1_ and the block hash values of *ϕ* successive blocks $\left\{bl_{t_{1}-\phi +1},bl_{t_{1}-\phi +2},\ldots ,bl_{t_{1}}\right\}$. Then the data owner outsources all the (*d*_*i*_,*s*_*i*_) pairs and the values $\left\{bl_{t_{1}-\phi +1},bl_{t_{1}-\phi +2},\ldots ,bl_{t_{1}},t_{1}\right\}$ to the CSP. Upon receiving these values, the storage server checks $e\left(s_{i},g\right)\overset{?}{=}e\left(g^{d_{i}},g\right)e\left(H\left(w\right),pk\right)$. If the verification passes, the log server stores $\left\{bl_{t_{1}-\phi +1},bl_{t_{1}-\phi +2},\ldots ,bl_{t_{1}},t_{1}\right\}$ and the storage server stores all the (*d*_*i*_,*s*_*i*_) pairs. Otherwise, the storage server rejects the values. Then, the storage server computes $\varphi =H(bl_{t_{1}-\phi +1}\|\ldots \|bl_{t_{1}}\|d_{1}\|s_{1}\|\ldots \|d_{m}\|s_{m})$. Then, the storage server generates a transaction *T*_2_ and sends it to the blockchain, where 0 ether is transferred from *A*_*P*_ to *A*_*C*_. The data value of the transaction *T*_1_ is set as *φ*.
- Query: Randomized auditing is adopted to audit the data integrity. The auditor generates random indexes (*i*_1_, *i*_2_, …, *i*_*l*_)∈{1, 2, …, *m*} with random values $\left(e_{i_{1}},e_{i_{2}},\ldots ,e_{i_{l}}\right)\;\;\in Z_{p}^{l}$. Then the auditor extracts the current block height *t*_2_ and the block hash values $\left\{bl_{t_{2}-\phi +1},bl_{t_{2}-\phi +2},\ldots ,bl_{t_{2}}\right\}$ from the blockchain and sends the values $\left\{bl_{t_{2}-\phi +1},bl_{t_{2}-\phi +2},\ldots ,bl_{t_{2}},t_{2}\right\}$ to the CSP. The auditor computes the hash value $\chi =H(bl_{t_{2}-\phi +1}\|\ldots \|bl_{t_{2}}\|t_{2})$. Subsequently, the auditor generates a transaction *T*_2_ to the blockchain with 0 ether transferring from *A*_*U*_ to *A*_*C*_. The data value of the transaction *T*_2_ is set as *χ*. At last, the auditor stores $\left\{bl_{t_{2}-\phi +1},bl_{t_{2}-\phi +2},\ldots ,bl_{t_{2}},t_{2}\right\}$ in the log file on the log server.
- Prove: The customized random mask is used to enhance the privacy-preserving property. The storage server randomly chooses $r\to Z_{p}$ and computes *θ* = *g*^*r*^, *ω* = *H*(*θ*), $\alpha^{\prime }=\sum_{i_{j}=1}^{l}d_{i_{j}}e_{i_{j}}$,*α* = *r*+ *ωα*′, and $\beta =\theta \prod_{i_{j}=1}^{l}s_{i_{j}}^{e_{i_{j}}}$. Afterward, the storage server returns back Γ = (*θ*, *α*, *β*) to the auditor as proof. Similarly, the storage server extracts the current block height *t*_3_ and the block hash values of *ϕ* successive blocks $\left\{bl_{t_{3}-\phi +1},bl_{t_{3}-\phi +2},\ldots ,bl_{t_{3}}\right\}$ from the blockchain. The storage server computes the value $\lambda =H(bl_{t_{3}-\phi +1}\|\ldots \|bl_{t_{3}}\|t_{3}\|\theta \|\alpha \|\beta )$ and sends a transaction *T*_3_, where 0 ether transferring from *A*_*C*_ to *A*_*U*_ and the data value of the transaction *T*_3_ is set as λ. Finally, the storage server stores the entry $\left\{bl_{t_{3}-\phi +1},bl_{t_{3}-\phi +2},\ldots ,bl_{t_{3}},t_{3},\theta ,\alpha ,\beta \right\}$ in the log file on the log server.
- Verify: Upon receiving the proof Γ = (*α*, *β*, *θ*), the auditor computes *ω* = *H*(*θ*) and checks whether $e\left(\beta ,g\right)\overset{?}{=}e\left(g^{\alpha },g\right)e\left(\prod_{i_{j}=1}^{l}H{\left(w\right)}^{e_{i_{j}}\omega },pk\right)$ If yes, the auditor accepts the proof and outputs the result *ξ* with the value of *True*. Otherwise, the auditor rejects the proof and outputs the result *ξ* with the value of *False*. Later on, the auditor extracts the current block height *t*_4_ and the block hash values $\left\{bl_{t_{4}-\phi +1},bl_{t_{4}-\phi +2},\ldots ,bl_{t_{4}}\right\}$ from the blockchain. The auditor computes $\varrho =H(bl_{t_{4}-\phi +1}\|\ldots \|bl_{t_{4}}\|t_{4}\|\xi )$ and sends a transaction *T*_4_, where 0 ether transferring from *A*_*U*_ to *A*_*P*_ and the data value of the transaction *T*_5_ is set as *ϱ*. At last, the log server stores the log entry $\left\{bl_{t_{4}-\phi +1},bl_{t_{4}-\phi +2},\ldots ,bl_{t_{4}},t_{4},\xi \right\}$ on the log server.

The phases’ results are recorded in the blockchain transactions and stored in the log server of CSP. Therefore, any third party or user can check for authenticity by leveraging the blockchain and the stored log entries of the CSP.

### 6.2 Correctness analysis

In what follows, the correctness proof for PPTPS is given.

**Theorem 1**: *If the corresponding entities follow under the steps in the phases of Delegate*, *Outsource*, *Query*, *Prove*, *and Verify strictly, the correctness of PPTPS can be demonstrated*.

According to Definition 1, we can demonstrate the algorithm Verify(*q*, Γ, *K*) outputs the result of *True*. We can demonstrate that the following Eq 1 holds.

$$\begin{array}{c} \begin{array}{c} e\left(\beta ,g\right)=e\left(g^{\alpha },g\right)e\left(\prod_{i_{j}=1}^{l}H{\left(w\right)}^{e_{i_{j}}\omega },pk\right) \end{array} \end{array}$$ (1)

The correctness proof is provided in the following.

$$\begin{array}{c} \begin{array}{cc} e(\beta ,g) & =e(\theta \prod_{i_{j}=1}^{l}s_{i_{j}}^{e_{i_{j}}\omega },g)\\ & =e(g^{r}\prod_{i_{j}=1}^{l}(g^{d_{i_{j}}}\cdot H(i_{j}{\left\|fn)\right)}^{e_{i_{j}}\omega },g)\\ & =e\left(g^{r}\prod_{i_{j}=1}^{l}g^{d_{i_{j}}e_{i_{j}}\omega },g\right)e(\prod_{i_{j}=1}^{l}H(i_{j}{\left\|fn\right)}^{e_{i_{j}}\omega },g^{x_{i_{j}}})\\ & =e\left(g^{r+d_{i_{j}}e_{i_{j}}\omega },g\right)e(\prod_{i_{j}=1}^{l}H(i_{j}{\left\|fn\right)}^{e_{i_{j}}\omega },pk)\\ & =e\left(g^{\alpha },g\right)e(\prod_{i_{j}=1}^{l}H(i_{j}{\left\|fn\right)}^{e_{i_{j}}\omega },pk). \end{array} \end{array}$$ (2)

## 7 Security analysis

We present a security analysis of PPTPS in three aspects. At first, we prove that the external adversary cannot forge the tag of the block through Theorem 2 and the malicious CSP cannot forge a valid proof on the corrupted data through Theorem 3. Then we give the security analysis for the procrastinating auditor and the malicious data owner, respectively. At last, we analyze the privacy-preserving property in the auditing query phase through Theorem 4.

**Theorem 2**: *If the adversary* $A_{I}$ *wins* $\text{Game}I(C,A_{I})$ *with the advantage ε, after making SecretKey Query, PublicKey Query, Hash Query, Tag Query at most* *q*_*sk*_, *q*_*pk*_, *q*_*H*_, *and* *q*_*T*_ *times respectively, then a simulator* B *can break the CDH problem with the non-negligible probability*.

*Proof*: Given a CDH instance $\left(g,G_{1},g^{a},g^{b}\right)$, if the adversary $A_{I}$ wins $\text{Game}I(C,A_{I})$ with non-negligible probability, the simulator B can calculate *g*^*ab*^ at non-negligible probability by the capability of $A_{I}$. B simulates each interaction step with $A_{I}$ as follows.

- Setup: B generates the system parameters.
- SecretKey Query: $A_{I}$ adaptively executes SecretKey Query Ṁeanwhile, B maintains a list *L*_1_ = (*pk*, *sk*, *T*).
  - If sk* does not exist in *L*_1_, B randomly chooses a value $x^{*}\in Z_{p}^{*}$ and flips a coin T* ∈ {0, 1}. Assume the probability of T* = 0 is *γ*, and the probability of T* = 1 is 1 − *γ*. If T* = 1, B calculates $pk={\left(g^{a}\right)}^{x^{*}}$ and adds the tuple (pk*, x*, T*) to *L*_1_. Then B aborts and outputs ‘fail’. If T* = 0, B calculates $pk=g^{x^{*}}$ and adds the tuple (pk*, x*, T*) to *L*_1_. Then B returns x* to $A_{I}$.
  - If sk* exists in *L*_1_, B checks T*. If T* = 1, B outputs ‘fail’ and abort. Otherwise, B retrieves sk* and returns it to $A_{I}$.
- PublicKey Query: $A_{I}$ adaptively executes PublicKey Query. Meanwhile, B maintains a list *L*_1_ = (*pk*, *sk*, *T*).
  - If the tuple (pk*, sk*, T*) already exists in *L*_1_, B returns pk* to $A_{I}$ directly.
  - If the list *L*_1_ does not contain the tuple (pk*, sk*, T*), B randomly selects a value $x^{*}\in Z_{p}^{*}$ and flips a coin T* ∈ {0, 1}. Assume the probability of T* = 0 is *γ*, and the the probability of T* = 1 is 1 − *γ*. For T* = 1, B calculates $pk^{*}=g^{x^{*}}$. B adds a new tuple (pk*, x*, T*) into *L*_1_ and returns back pk* to $A_{I}$.
- Hash Query: $A_{I}$ adaptively executes Hash Query for $w\in Z_{p}^{*}$. B also maintains a list *L*_2_ = {*w*, *h*}. If the list *L*_2_ already contains w*, B retrieves h* and returns ${\left(g^{b}\right)}^{h^{*}}$ to $A_{I}$. Otherwise, B selects a random value $h\in Z_{p}^{*}$ and returns ${\left(g^{b}\right)}^{h^{*}}$ to $A_{I}$. Finally B inserts (w*, h*) into the list *L*_2_.
- Tag Query: $A_{I}$ adaptively executes Tag Query with (w*, d*). At first, B checks whether T* = 0 in the list *L*_1_. If T* = 0, B gets sk* from *L*_1_ and *H*(w*) from *L*_2_. Otherwise, B aborts and outputs ‘fail’. Then B computes the tag for (w*, d*) by the algorithm Outsource and returns the tag to $A_{I}$.
- Forge: $A_{I}$ outputs a tuple *O* = (*s*′, *w*′, *d*′). *s*′ is a forged tag on the block *d*′.
- Analysis: If $A_{I}$ wins $\text{Game}I(C,A_{I})$, B cam get $e\left(s^{\prime },g\right)=e\left(g^{d^{\prime }},g\right)e\left(H\left(w^{\prime }\right),pk^{\prime }\right)$, Then B retrieves the tuple (*pk*′, *x*′, *T*′) from *L*_1_. If T* = 0, B aborts and outputs ‘fail’. Otherwise, B gets $pk^{\prime }=g^{ax^{\prime }}$ from *L*_1_ and $H\left(w^{\prime }\right)=g^{bh^{\prime }}$ from *L*_2_. Then B gets $e\left(s^{\prime },g\right)=e\left(g^{d^{\prime }},g\right)e\left(g^{bh^{\prime }},g^{ax^{\prime }}\right)$. We get $g^{ab}={\left(s^{\prime }\right)}^{\frac{1}{x^{\prime }h^{\prime }d^{\prime }}}$. Therefore, the probability that B simulates the interactions with $A_{I}$ without abortion is higher than ${\left(1-\gamma \right)}^{q_{sk}+q_{T}}$. B outputs *g*^*ab*^ with the probability $\varepsilon^{\prime }\geq \varepsilon \gamma {\left(1-\gamma \right)}^{q_{s}k+q_{T}}\geq \frac{\varepsilon }{2e(q_{s}k+q_{T})}$. Therefore, B breaks the CDH problem with the non-negligible probability.

**Theorem 3**: *If security for DLP is guaranteed, then the probability that the adversary* $A_{II}$ *wins* $\text{Game}II(C,A_{II})$ *is negligible*.

*Proof*: If $A_{II}$ outputs the integrity proof Γ_1_ = (*θ*, *α*_1_, *β*_1_) and wins $\text{Game}II(C,A_{II})$ with non-negligible probability, we can get Eq 3.

$$\begin{array}{c} \begin{array}{c} e\left(\beta_{1},g\right)=e\left(g^{\alpha_{1}},g\right)e(\prod_{i_{j}=1}^{l}H(i_{j}{\left\|fn\right)}^{e_{i_{j}}\omega },pk). \end{array} \end{array}$$ (3)

Because $A_{II}$ wins $\text{Game}II(C,A_{II})$, there exits *β*_1_ = *β* but *α*_1_ ≠ *α*. According Eqs 1 and 3, we define Δ*α* = *α*_1_ − *α* and get *g*^Δ*α*^ = 1. Given $h,u\in G_{1}$, we set *u* = *h*^*x*^ and compute $x\in Z_{p}^{*}$. Let *g* = *h*^*a*^ *u*^*b*^ where *b* is randomly chosen and $a,b\in Z_{p}^{*}$, we get (*h*^*a*^ *u*^*b*^)^Δ*α*^ = 1 and *u* = *h*^−*a*/*b*^. Since $b\in Z_{p}^{*}$ is randomly chosen and information-theoretically hidden from the adversary [39], we get the right value *x* and solve DLP with a non-negligible probability 1 − 1/*q*. To resist attacks from the internal adversaries in Section 5.3, we describe two security properties, respectively.

### 7.1 PPTPS guarantees the timeliness

In each phase, we integrate the corresponding information into a blockchain transaction. Hence, the block hashes and the block height in which the transaction is integrated reflect the timeliness of PPTPS. After the transaction is recorded, any third party can extract the corresponding block hashes and the block height. For example, in the Verify phase, the block height is *t*_3_. the user gets the log entry $\left\{bl_{t_{3}-\phi +1},bl_{t_{3}-\phi +2},\ldots ,bl_{t_{3}},t_{3},\theta ,\alpha ,\beta \right\}$ stored on the storage server and computes $\lambda^{\prime }=H(bl_{t_{3}-\phi +1}\|\ldots \|bl_{t_{3}}\|t_{3}\left\|\theta \|\alpha \|\beta \right)$. Besides, the security of timeliness is guaranteed by the underlying blockchain. To our best knowledge, if an adversary is without a 51% hash rate, it cannot break the security of the blockchain. Thus, timeliness security is well guaranteed.

### 7.2 Resistance against the procrastinating auditor

PPTPS can resist malicious auditors. First, since the security of PPTPS has been demonstrated in the game Game(C,A), it is computationally infeasible for a malicious auditor to generate an entry $\left\{bl_{t_{4}-\phi +1},bl_{t_{4}-\phi +2},\ldots ,bl_{t_{4}},t_{4},\theta ,\alpha ,\beta \right\}$ that passes the verification in the Verify phase. Second, Blockchain security guarantees timeliness. For the procrastinating auditor, it is computationally infeasible to generate a transaction at the block height *t*_2_. Still, the malicious auditor convinces others that the transaction is generated at the block height *t*_1_.

### 7.3 Privacy-preserving audit

The below Theorem 4 shows that the auditor cannot derive the data owner’s data from information generated during auditing.

**Theorem 4**: *From the CSP’s response* Γ = (*θ*, *α*, *β*), *the auditor cannot recover* *α*′.

**Proof**. Without random masking, the CSP computes $\alpha =\sum_{i_{j}=1}^{l}d_{i_{j}}e_{i_{j}}$, $\beta =\prod_{i_{j}=1}^{l}s_{i_{j}}^{e_{i_{j}}}$ and sends Γ = (*α*, *β*) as the proof. However, in this way, there exists potential information leakage. Since by collecting a sufficient number of linear combinations of the same set of data blocks, the auditor can generate a group of linear equations and solve these equations to derive the data owner’s data. However, with random masking, the CSP computes *θ* = *g*^*r*^, *ω* = *H*(*θ*), $\alpha^{\prime }=\sum_{i_{j}=1}^{l}d_{i_{j}}e_{i_{j}}$,*α* = *r*+ *ωα*′, $\beta =\theta \prod_{i_{j}=1}^{l}s_{i_{j}}^{e_{i_{j}}}$ and sends Γ = (*θ*, *α*, *β*) as the proof. Straightforwardly, due to the security properties of the random number and the hash function, the auditor cannot recover *α*′. Therefore, the auditor cannot derive the data owner’s data.

## 8 Experimental evaluation

By conducting extensive experiments, we evaluate the performance of PPTPS in computation and communication costs. The experiments are performed on a Win7 operating system, an Intel Core 2 i5 CPU, and 8 GB DDR 3 RAM. Java Development Kit and Eclipse IDE for Java Developers are installed. We use java.security, java.math, and java.util packages to evaluate the computation and communication costs. Besides, all the EHRs datasets as benchmarks are public, which can be downloaded from [40]. The sizes of the selected five benchmarks range from 6KB to 13MB. The block size is set as 1024 bits, the number of successive blocks is set as *ϕ* = 12, and the number of challenged blocks is set as 30 by default. We run ten times for each experiment and take the average value as the final result for more precision.

### 8.1 Computation cost

**Fig 2:**
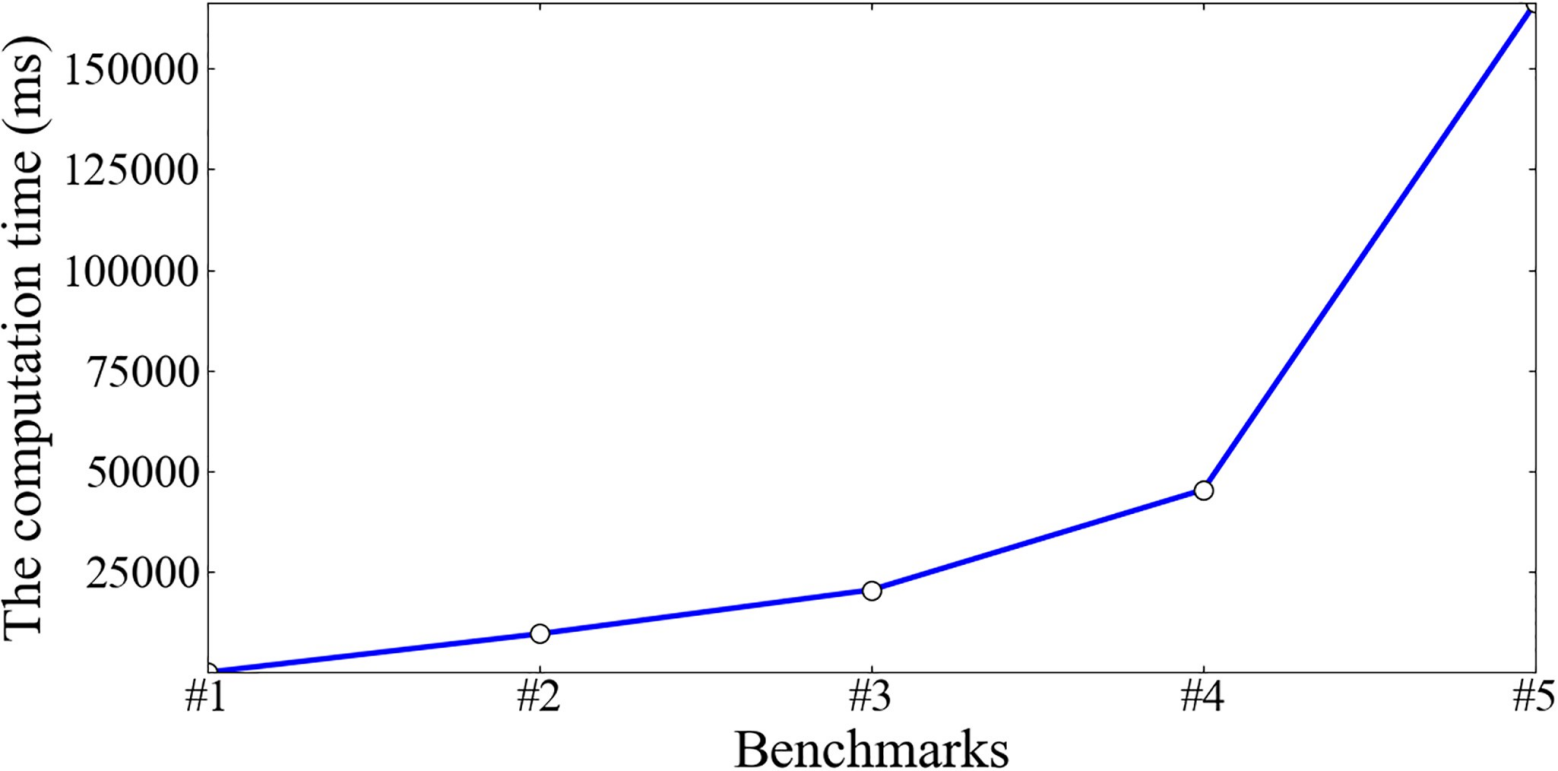
The computation costs in the Outsource phase (ms).

**Fig 3:**
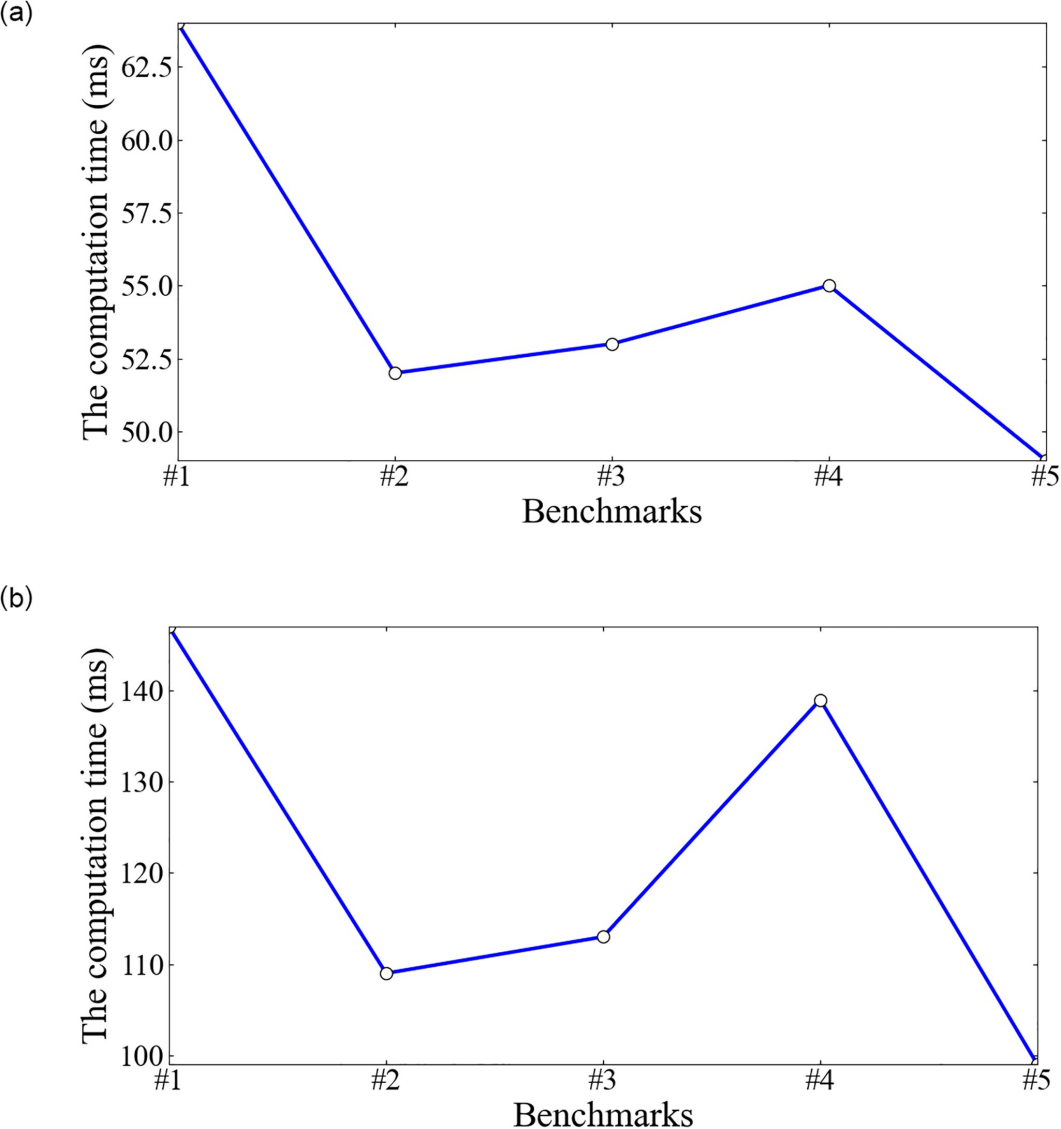
The computation costs in the Prove and Verify phases (ms).

We observe the computation cost in each phase. In the Delegate and Query phase, the operations need few computation costs. Hence, we focus on the computation costs in the Outsource, Prove, and Verify phases. In Figs 2 and 3, it is observed that the computation costs in the phases of Prove and Verify vary slightly, within 1s with the increment of benchmark sizes. However, the computation cost in the Outsource phase increases dramatically with the increment of benchmark sizes.

In the following, we compare the computation cost with previous works. The basic cryptographic operations and their meanings of the computation cost are listed in Table 2. Then we compare the computation costs of the CSP side with two previous works, SWP [11] and CPVPA [8]. In PPTPS, it mainly derives from the Outsource phase and the Prove phase, where the CSP checks the tags’ validity and the CSP generates the integrity proof, respectively. Let *l* denote the challenged data blocks, according to the equations *θ* = *g*^*r*^, *ω* = *H*(*θ*), $\alpha =r+\omega \sum_{j=1}^{l}d_{i_{j}}e_{i_{j}}$, $\beta =\prod_{j=1}^{l}s_{i_{j}}^{e_{i_{j}}}$, and $\lambda =H(bl_{t_{4}-\phi +1}\|\ldots \|bl_{t_{4}}\|t_{4}\|\theta \|\alpha \|\beta )$, we obtain that there are *l* ⋅ *Exp*_*G*_ operations, (*l* − 1) ⋅ *Mul*_*G*_ operations, $l\cdot Mul_{Z_{p}}$ operations, $l\cdot Add_{Z_{p}}$, and $2\cdot H_{Z_{p}}$ operations of the CSP side. It is shown in Table 3 that the computation cost in PPTPS is less than the computation cost in CPVPA and slightly more than the computation cost in SWP. Compared to SWP, the computation increment leads PPTPS to possess privacy-preserving and traceable properties. Therefore, this computation increment can be tolerated in practice.

**Table 2:** Cryptographic notations.

Notations	Meanings
$Exp_{G}$	exponentiation operation in G
$Mul_{G}$	multiplication in G
$Mul_{Z_{p}}$	multiplication in $Z_{p}$
*PRF*	permutated random function
$Add_{Z_{p}}$	addition in $Z_{p}$
$Pair_{G}$	pairing operation in G
$H_{G}$	hash a value into G
$H_{Z_{p}}$	hash a value into $Z_{p}$

**Table 3:** The computation cost of the CSP side.

Schemes	The computation cost of the CSP side
SWP [11]	$l\cdot Exp_{G}+l\cdot Mul_{G}+l\cdot Mul_{Z_{p}}+l\cdot Add_{Z_{p}}$
CPVPA [8]	$l\cdot Exp_{G}+l\cdot Mul_{G}+l\cdot Mul_{Z_{p}}+l\cdot Add_{Z_{p}}+2l\cdot PRF$
CPIGS [38]	$3\cdot Pair_{G_{T}}+m\cdot H_{G}+m\cdot H_{Z_{p}}+2l\cdot PRF+\left(l+m\right)\cdot Exp_{G}+l\cdot Mul_{G}+l\cdot Mul_{Z_{p}}+l\cdot Add_{Z_{p}}$
PPTPS	$3\cdot Pair_{G_{T}}+m\cdot Exp_{G}+m\cdot Hash_{G}+l\cdot Exp_{G}+\left(l-1\right)\cdot Mul_{G}+l\cdot Mul_{Z_{p}}+l\cdot Add_{Z_{p}}+2\cdot H_{Z_{p}}$

**Fig 4:**
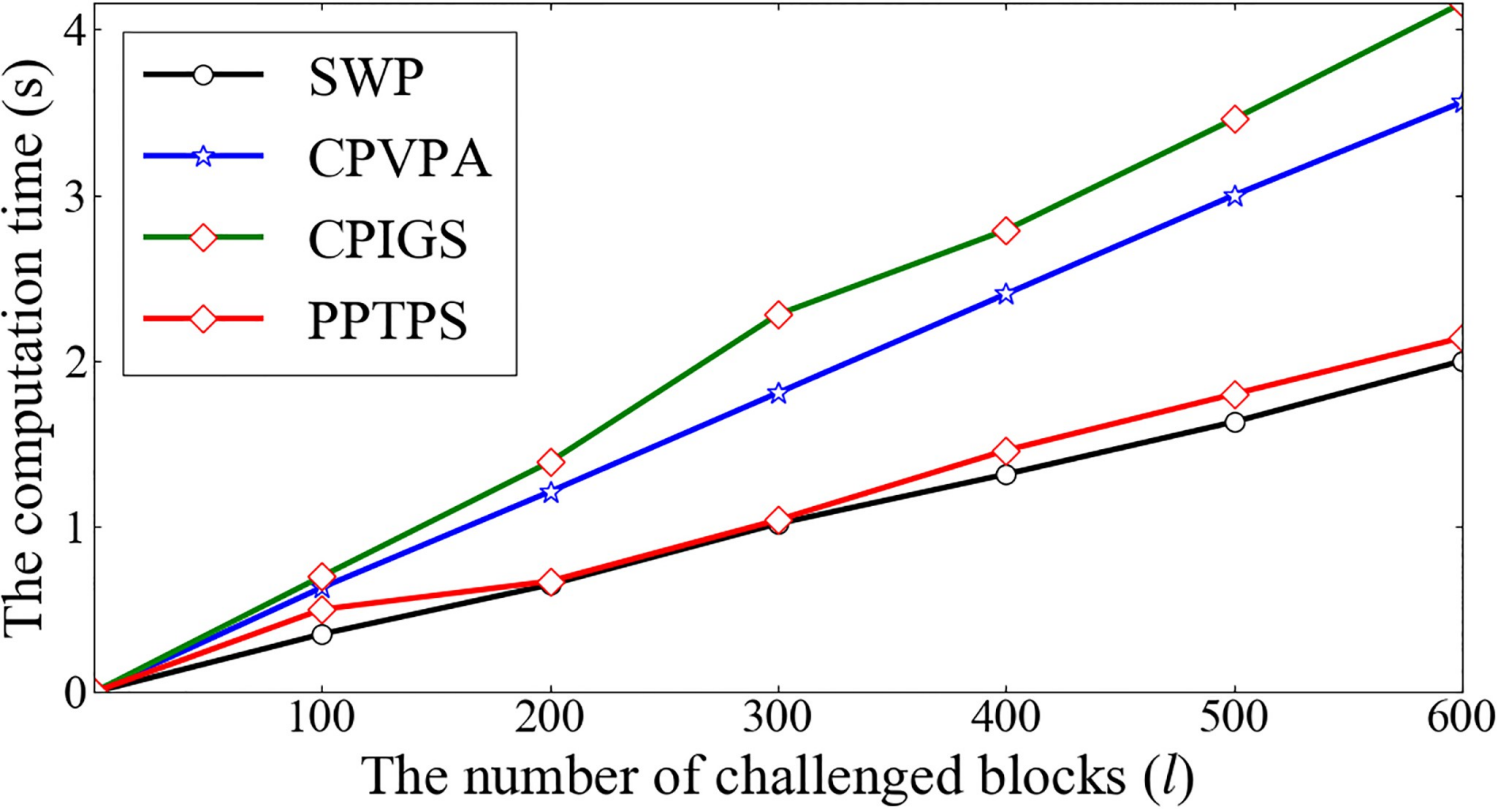
The computation cost of the auditor side.

Moreover, the computation cost of the auditor side derives from the Verify phase. We compute *ω* = *H*(*θ*), $\lambda =H(bl_{t_{5}-\phi +1}\|\ldots \|bl_{t_{5}}\|t_{5}\|\xi )$, and checks whether $\beta \overset{?}{=}g^{\alpha }\cdot \prod_{j=1}^{l}y_{i_{j}}^{e_{i_{j}}\omega }\cdot \prod_{j=1}^{l}H(i_{j}{\left\|fn\right)}^{e_{i_{j}}\omega }$, where $\beta =\prod_{i_{j}=1}^{l}s_{i_{j}}^{e_{i_{j}}}$. Therefore, there are (3*l* + 1) ⋅ *Exp*_*G*_ operations, (3*l* − 1) ⋅ *Mul*_*G*_ operations, $\left(l+2\right)\cdot H_{Z_{p}}$ operations in this phase. The computation cost of the auditor side is described in Table 4. It is observed that the computation cost in PPTPS is no more than the computation costs in SWP and CPVPA. Fig 4 further demonstrates that PPTPS does not increase the computation cost. In the case of 200 challenged blocks, the auditor verifies the proof within 1 minute.

**Table 4:** The computation cost of the auditor side.

Schemes	The computation cost of the auditor side
SWP [11]	$2\cdot Pair_{G_{T}}+\left(l+1\right)\cdot Exp_{G}+l\cdot Mul_{G}+l\cdot Hash_{G}$
CPVPA [8]	$4\cdot Pair_{G_{T}}+\left(3l+2\right)\cdot Exp_{G}+3l\cdot Mul_{G}+\left(l+4\right)\cdot H_{G}+\left(2l+3\right)\cdot H_{Z_{p}}+2l\cdot Mul_{Z_{p}}+2l\cdot PRF$
CPIGS [38]	$3\cdot Pair_{G_{T}}+2l\cdot PRF+2l\cdot H_{G}+2l\cdot Exp_{G}+3l\cdot Mul_{G}$
PPTPS	$3\cdot Pair_{G_{T}}+\left(3l+1\right)\cdot Exp_{G}+\left(3l-1\right)\cdot Mul_{G}+l\cdot H_{G}+2\cdot H_{Z_{p}}$

**Fig 5:**
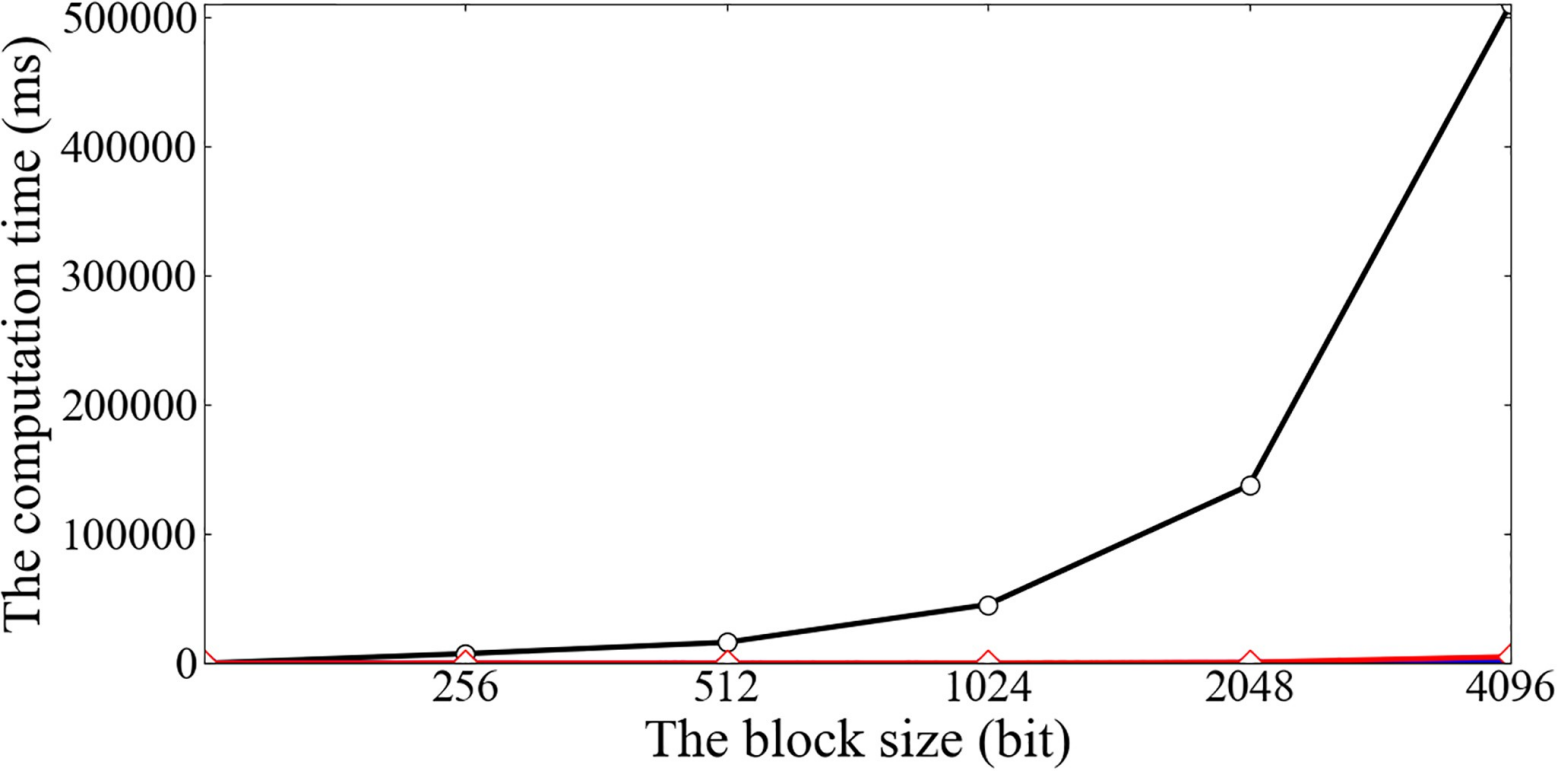
The computation cost for different block sizes.

Finally, we vary the block size from 256 bits to 4096 bits. It is shown in Fig 5 that the computation costs in the Outsource, Verify, and Prove phases increase with the increment of the block size. Besides, the computation cost in the Outsource phase increases most dramatically. Note that the longer the block size is, the smaller the block number. Therefore, a tradeoff is made between the block size and the block number in pursuit of high efficiency.

### 8.2 Communication cost

**Fig 6:**
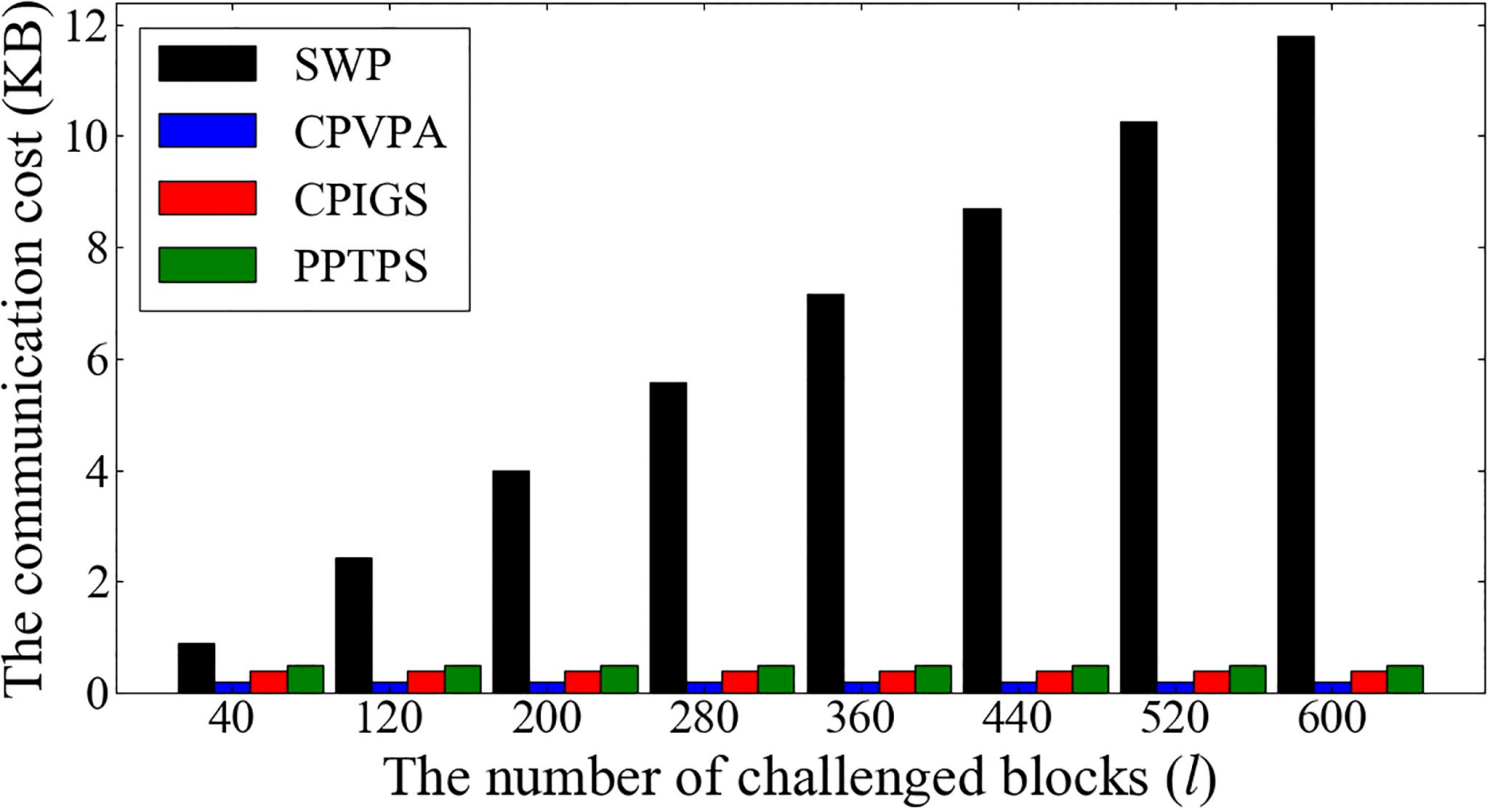
The communication cost between the CSP and the auditor.

The communication cost involves two types. One type of communication cost derives from transferring the generated proof Γ = (*θ*, *α*, *β*) between the CSP and the auditor, and the other type of communication cost derives from sending the generated hash value in each phase from the relevant party to blockchain. As to the former one, the same as in CPVPA [8], it is irrelevant to the challenged blocks and keeps constant. It is shown in Fig 6 that the communication cost between the CSP and the auditor is about 0.5KB. Therefore, it is superior to the communication cost in SWP [11]. The latter type of communication cost comprises the hash value *φ*, *χ*, λ, and ϱ. We use Solidity’s data type of ‘uint256’ to store these hash values. Therefore, the communication cost in this part is 128 bytes.

### 8.3 monetary cost

In PPTPS, the monetary costs are caused by conducting the corresponding transaction in Ethereum in each phase. For example, once a user generates and outsources data to the CSP, the user creates a transaction *T*_1_. Next, an auditor generates an audit query and creates a transaction *T*_2_ in the query phase. moreover, the CSP generates a proof and creates a transaction *T*_3_ in the proving phase. At last, the auditor verifies the proof and renders the result. Then the auditor creates a transaction *T*_4_ to record the verification result.

Since each transaction on Ethereum needs computational effort to execute, each transaction requires a fee. In addition, Gas refers to the fee which is required to execute the specific operations on Ethereum. We compute the cost fee by (cost Gas) × (gas price). The gas price is set as 2 gwei, where 1 gwei = 10^−9^ ETH. Among all the transactions *T*_1_ − *T*_4_, the most gas costed by *T*_3_ is 644169 gas. When writing this paper (September 21, 2022), 1 ETH is equivalent to $1430. Then we can observe that the most expensive transaction cost is about $1.84. Thus, the entire monetary cost for all the transactions is about $7.36, which is realistically affordable.

### 8.4 Discussion

PPTPS can be widely used in the field of public cloud storage. The timeliness enables PPTPS to keep track of critical activities for cloud storage, such as Outsource, Audit, Prove, and Verify. However, there are two necessary assumptions for PPTPS to work.

**Assumption 1**. The data owner does not collude with the CSP. moreover, the auditor is assumed to be a trusted third party and does not collude with the data owner and the CSP.

**Assumption 2**. PPTPS assumes that the transactions are all valid after posting to the blockchain. Miners in Ethereum are responsible for validating the transactions and packaging the valid transactions into Ethereum.

## 9 Conclusion and future work

In this paper, we construct a privacy-preserving publicly-auditable cloud storage system and present a traceable management method for the cloud storage system. Then we formally prove the security of PPTPS and analyze the resistance against different threats in detail. Finally, the experimental evaluation demonstrates its feasibility in computation, communication, and monetary cost. In the future, we will explore how to integrate blockchain with current cloud storage systems in depth, e.g., introducing the smart contract to provide more complicated and intelligent management for cloud storage systems. moreover, we will focus on the auditing problem based on searchable encryption in the follow-up work [41].
